# Patient-perceived benefits and risks of off-label use of SGLT2 inhibitors and GLP-1 receptor agonists in type 1 diabetes: a structured qualitative assessment

**DOI:** 10.1177/20420188231180987

**Published:** 2023-06-29

**Authors:** Khary Edwards, Aleksandra Uruska, Anna Duda-Sobczak, Dorota Zozulinska-Ziolkiewicz, Ildiko Lingvay

**Affiliations:** Department of Internal Medicine/Endocrinology, University of Texas Southwestern Medical Center, 5323 Harry Hines Blvd, Dallas, TX 75390-8857, USA; Department of Internal Medicine and Diabetology, Poznan University of Medical Sciences, Poznan, Poland; Department of Internal Medicine and Diabetology, Poznan University of Medical Sciences, Poznan, Poland; Department of Internal Medicine and Diabetology, Poznan University of Medical Sciences, Poznan, Poland; Department of Internal Medicine/Endocrinology, Department of Population and Data Sciences, University of Texas Southwestern Medical Center, Dallas, TX, USA

**Keywords:** GLP-1RA, patient-reported outcomes, SGLT2i, T1DM

## Abstract

**Background:**

Patients with type 1 diabetes mellitus (T1DM) may have suboptimal glucose control and are interested in the use of adjuvant therapies.

**Objectives:**

To determine, from the patients’ perspective, the reasons for initiation of glucagon-like peptide 1 receptor agonist (GLP-1RA) and/or sodium glucose cotransporter 2 inhibitor (SGLT2i) in treating T1DM; perceived benefits/side effects, reasons for discontinuation, and willingness to reinitiate therapy.

**Design:**

Retrospective chart review with structured telephone interviews.

**Methods:**

We identified patients with T1DM treated with a GLP-1RA and/or SGLT2i for >3 months at University of Texas Southwestern Medical Center (Dallas, TX, USA) and Poznan University (Poznan, Poland). We conducted structured telephone interviews regarding their experiences.

**Results:**

We interviewed 68 participants treated with GLP-1RA and 82 with SGLT2i. Treatment was initiated for improving glycemic control (as reported by 61.8% *versus* 81.7% of GLP-1RA and SGLT2i users, respectively), weight loss/appetite suppression (51.4% *versus* 23.2%) and to reduce insulin requirement (13.2% *versus* 11%). Most participants (86.8% of GLP-1RA and 89.0% of SGLT2i users) reported ⩾1 benefit attributed to therapy. Reported benefits were improved glycemic control (reported by 58.8% *versus* 82.9% of GLP-1RA and SGLT2i users, respectively), weight loss/appetite suppression (63.2% *versus* 30.5%), and reduced insulin requirement (27.9% *versus* 34.1%). More GLP-1RA users reported side effects *versus* SGLT2i users (63.2% *versus* 36.6%); 22.6% discontinued GLP-1RA due to side effects *versus* 11.0% SGLT2i users. Diabetic ketoacidosis (DKA) was reported by 4.9% of SGLT2i users, but none in GLP-1RA users. Of those who discontinued medication, 60.7% of GLP-1RA *versus* 56.0% of SGLT2i prior users were willing to reinitiate treatment.

**Conclusions:**

Patients with T1DM report initiating adjuvant treatment with GLP-1RA and/or SGLT2i to improve glycemic control and lose weight; most patients reported perceived benefits from these therapies. Side effects (including DKA) are reported more commonly in real life than in clinical trials. Given patient interest in these medications, further studies should evaluate the long-term risk–benefits ratio in larger cohorts.

## Introduction

Type 1 diabetes mellitus (T1DM) was a fatal disease before the discovery of insulin in 1921,^1^ which has become the mainstay of treatment ever since. While treatment with insulin is life-preserving for patients with T1DM, opportunities remain to optimize treatment, especially regarding fine-tuning glycemic control and managing cardiovascular risk.

Glucagon-like peptide 1 receptor agonists (GLP-1RAs)^2,3^ and sodium glucose cotransporter 2 inhibitors (SGLT2i)^4^ have been shown to improve glucose control, lead to weight loss, and reduction in cardiovascular and renal events in patients with type 2 diabetes mellitus (T2DM), and therefore current treatment guidelines widely recommend their use in those with T2DM. Agents from both these classes were also evaluated in those with T1DM.^5,6^ Preliminary studies with GLP-1RAs as adjunct therapy for T1DM showed beneficial effects on glycemic control and weight management, but further development was stopped due to a concern for potentially increasing the risk for hypoglycemic events.^7,8^ Several SGLT2i (dapagliflozin, empagliflozin, and sotagliflozin – the latter being a dual SGLT1 and SGLT2i) were evaluated in full phase III programs^9–12^ and have also shown modest benefits on glycemic control and weight but were also associated with an increased risk of diabetic ketoacidosis (DKA). As a result of the concern for DKA coupled with the relatively modest efficacy, the U.S. Food and Drug Administration has not approved any of these agents for use in patients with T1DM. The European Medicines Agency initially approved dapagliflozin and sotagliflozin specifically for patients with T1DM with a body mass index (BMI) >27 kg/m^2^ in 2019. Subsequently, both agents had this indication voluntarily withdrawn, with dapagliflozin withdrawn in October 2021 and sotagliflozin in May 2022, with concerns potentially relating to DKA risk.^13,14^ No studies to date evaluated the effect of these agents on cardiovascular events in patients with T1DM.

Nevertheless, both SGLT2i and GLP-1RAs are used off label in real-world clinical practice to manage T1DM. It was estimated that in 2016 in the United States approximately 13% of those with T1DM over the age of 26 years were using an adjuvant therapy in addition to insulin; 3% were using a SGLT2i and 2.5% a GLP-1RA.^15^ Most recently, our group published a work showing the real-world benefit of these medications in T1DM patients where after 1 year of therapy, GLP-1RA users had significant reductions in weight, hemoglobin A1c (HbA1c), and total daily dose of insulin, while SGLT2i users experienced significant reductions in HbA1c and basal insulin requirements.^16^

While our recent work offered important objective information on the use of these therapies, it is important to understand what drives their off-label use in clinical practice as well as assess patients’ perceptions regarding their benefits, side effects, and willingness to be treated with these agents in addition to an already complicated insulin regimen. In this current study, we investigated the patient-perceived reasons for initiation, perceived benefits, side effects experienced, and willingness to continue or restart adjuvant treatment with GLP-1RA or SGLT2i in patients with T1DM. Telephone interviews were conducted with patients at two large academic clinical practices – one in the United States and one in Europe.

## Materials and methods

We conducted a retrospective chart review to identify adults (age > 18 years) with T1DM ever treated with GLP-1RA and/or SGLT2i for at least 90 days at two academic centers: University of Texas Southwestern (UT Southwestern) Medical Center, Dallas, TX, USA and the Department of Internal Medicine and Diabetology, Poznan University of Medical Sciences, Poznan, Poland.

Patients with T1DM were identified by electronic query of the Electronic Medical Record based on *International Classification of Diseases, ninth revision (ICD-9) and 10th revision (ICD-10)* codes (ICD-9 250.x1, 250.x3; ICD-10 E10.xx) and selected if they ever received a prescription for either a GLP-1RA or a SGLT2i. Resulting charts were manually reviewed to confirm accurate diagnosis of T1DM and select those in whom confirmation could be obtained from clinical notes that treatment occurred for a minimum of 90 days. The diagnosis of T1DM was confirmed by reviewing all clinical notes, documentation of chronic insulin use, and, where available, the presence of supporting laboratory studies (C-peptide and T1DM-related autoantibodies). We extracted demographic data (age at index date, sex, race, and ethnicity) and actual start and stop dates of the adjuvant therapy.

Patients who met eligibility criteria were contacted through telephone to conduct the survey. Diagnosis of T1DM and use of adjuvant therapy was confirmed during the interview. The following questions were asked:

1. What were the main reasons you decided to start taking [insert medication name of GLP-1RA/SGLT-2i]?
2. What were the top benefits (if any) you believe you gained from using this medication?
3. Did you experience any side effects that you or your doctor thought were related to this medication?
3a: If yes: What side effects and how long did they last?
4. If you are no longer taking [insert medication name], why did you stop taking it?
5a. Would you ever consider trying a similar treatment (same medication or another medication from the same class)?
5b:  If yes: Why? If no: Why not?

Responses were documented during the interview and then grouped into predefined categories. Multiple answers were allowed where applicable. At the UT Southwestern site, only one person conducted surveys to ensure consistency in the way questions were posed and responses were recorded. At Poznan University, two people conducted interviews, but worked together initially, again to ensure consistency in how questions were asked and responses were recorded. Three attempts were made to contact all the eligible patients. All interviews were conducted between November 2021 and February 2022.

The final population included patients with T1DM, 18 years or older at the time of the first use, who were treated with an adjuvant agent continuously for at least 90 days, and consented to participate in the survey. Patients in whom the diagnosis of T1DM could not be confirmed, who denied having T1DM, or denied ever using either medication were excluded.

Data were recorded in REDCap.^17^ We report descriptive analyses for the baseline characteristics and each response as proportion of the eligible population. Data are presented by treatment group (GLP-1RA and SGLT2i) in the overall cohort as well as within the cohort of each institution (UT Southwestern and Poznan University).

## Ethical procedures

This project was approved by the Institutional Review Boards of the UT Southwestern and Poznan University of Medical Sciences (reference STU 2021-0145). Informed consent was waived for the retrospective chart review and identification of patients who met the criteria for survey participation. At the time of telephone survey, participants provided verbal consent to the telephone interview prior to the survey questions being asked. Participants were made aware that if any question made them uncomfortable, they could rescind consent at any time during the survey and results would not be used or published. Participants were aware that reported survey information would be anonymous. Verbal consent was recorded in REDCap by the surveyor.

## Results

### Baseline characteristics

Overall, 266 eligible participants (196 at the US site and 70 at the Polish site) were identified from the medical record search. At the US site, contact was made with 116/196 patients of which 91 participants agreed to complete the survey. At the Polish site, 47/70 eligible participants agreed to complete the survey. Overall, 138 participants were surveyed (a positive response rate of 51.9%). Of these participants, 12 (10 in the US cohort and 2 in the Polish cohort) used both a GLP-1RA and a SGLT2i. These participants completed two surveys, one for each class. The total number of surveys completed was 150: 68 for GLP-1RA and 82 for SGLT2i.

The baseline characteristics of the overall cohort as well as the subgroup from each institution are presented in Table 1. The mean (±standard deviation) age (45 ± 13 years) was the same across both treatment groups. While the gender distribution was similar across the institutions, GLP-1RA users were more likely to be female (73.5% *versus* 54.9% for SGLT2i users). Most participants were White (all participants in Poland; 85.5% of GLP-1RA users and 79.4% of SGLT2i users in the United States) and non-Hispanic (all participants in Poland; 91.9% of GLP1-1RA users and 100% of SGLT2i users in the United States); the few representing minority populations in the US cohort were equally distributed in the two treatment groups. Median interquartile range duration of adjuvant treatment was 24.0 (30.5) and 15.7 (24.4) months for GLP-1RA and SGLT2i, respectively, and more than twice as long in the United States compared to Poland. Notably, there were very few GLP-1RA users in the Polish cohort.

**Table 1.** Patients’ baseline characteristics at the time of initiation of adjuvant therapy for type 1 diabetes.

Baseline characteristics	Total cohort		US cohort		Polish cohort	
	GLP-1RA (*N* = 68)	SGLT2i (*N* = 82)	GLP-1RA (*N* = 62)	SGLT2i (*N* = 39)	GLP-1RA (*N* = 6)	SGLT2i (*N* = 43)
Sex [*n* (%)]						
Female	50 (73.5)	45 (54.9)	44 (71.0)	20 (51.3)	6 (100)	25 (58.1)
Male	18 (26.5)	37 (45.1)	18 (29.0)	19 (48.7)	0 (0)	18 (41.9)
Age, years (mean ± SD)	45 ± 13	45 ± 13	45 ± 13	48 ± 13	48 ± 16	42 ± 12
Duration of therapy, months [median (IQR)]	24.0 (30.5)	15.7 (24.4)	30.0 (30.7)	24.1 (36.4)	8.0 (15.5)	12.0 (18.0)
Race [*n* (%)]						
White	59 (86.8)	74 (90.2)	53 (85.5)	31 (79.4)	6 (100)	43 (100)
Black or African American	6 (8.8)	5 (6.1)	6 (9.7)	5 (12.8)	0 (0)	0 (0)
Asian	1 (1.5)	3 (3.7)	1 (1.6)	3 (7.7)	0 (0)	0 (0)
Ethnicity [*n* (%)]						
Non-Hispanic	63 (92.6)	82 (100)	57 (91.9)	39 (100)	6 (100)	43 (100)
Hispanic	4 (5.9)	0 (0.0)	4 (6.5)	0 (0)	0 (0)	0 (0)

GLP-1RA, glucagon-like peptide 1 receptor agonist; IQR, interquartile range; *n*, number of participants in respective group; SD, standard deviation; SGLT2i, sodium glucose cotransporter 2 inhibitor.

### Self-reported reasons for initiation of adjuvant therapy

**Figure 1.**
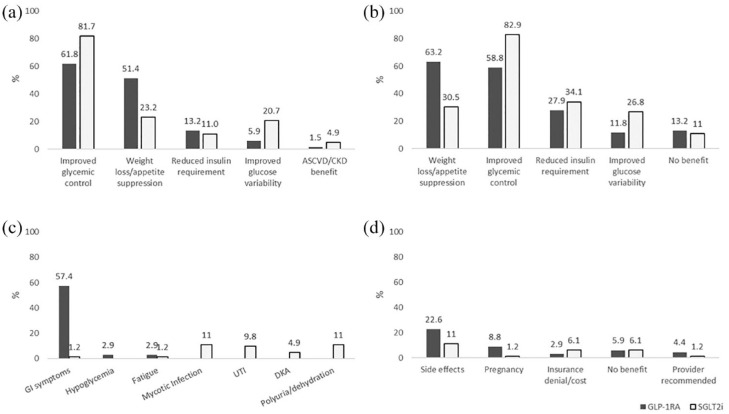
Survey responses regarding reasons for initiation, perceived benefits, side effects, and reasons for discontinuation for the entire cohort. (a) Reason(s) for initiation of therapy, (b) perceived benefit(s) attributed to treatment, (c) side effect(s) perceived to be related to therapy, and (d) reason(s) for therapy discontinuation. ASCVD, atherosclerotic cardiovascular disease; CKD, chronic kidney disease; DKA, diabetic ketoacidosis; UTI, urinary tract infection.

Survey results by treatment group and institution are presented in Table 2. The top reasons for initiating treatment with GLP-1RA were improved glycemic control (61.8%) and weight loss/appetite suppression (51.4%), while in those treated with SGLT2i the majority indicated starting therapy for improved glycemic control (81.7%), weight loss/appetite suppression (23.2%), or improved glucose variability (20.7%) (Figure 1(a)). Among SGLT2i users, those from the Polish cohort were more likely to report weight loss/appetite suppression (34.9% *versus* 10.3% for the Polish *versus* US cohorts) or decrease in insulin requirement (16.3% *versus* 5.1%) as reason for initiation, while those in the US cohort were more likely to report decrease in glucose variability as a reason for initiating therapy (30.8% *versus* 11.7%, for the US *versus* Polish cohorts) (Table 2)

**Table 2.** Survey responses regarding patients’ experience with adjuvant therapy for type 1 diabetes.

Question(s) and patient response(s)^a^ [*n*/*N* (%)]	Total cohort		US cohort		Polish cohort	
	GLP-1RA	SGLT2i	GLP-1RA	SGLT2i	GLP-1RA	SGLT2i
Reason(s) for initiation						
Improved glycemic control	42/68 (61.8)	67/82 (81.7)	37/62 (59.7)	30/39 (76.9)	5/6 (83.3)	37/43 (86.0)
Weight loss/appetite suppression	35/68 (51.4)	19/82 (23.2)	33/62 (53.2)	4/39 (10.3)	2/6 (33.3)	15/43 (34.9)
Reduced insulin requirement	9/68 (13.2)	9/82 (11.0)	8/62 (12.9)	2/39 (5.1)	1/6 (16.7)	7/43 (16.3)
Improved glucose variability	4/68 (5.9)	17/82 (20.7)	3/62 (4.8)	12/39 (30.8)	1/6 (16.7)	5/43 (11.7)
ASCVD/CKD benefit	1/68 (1.5)	4/82 (4.9)	1/62 (1.6)	4/39 (10.3)	0/6 (0.0)	0/43 (0.0)
Perceived benefit(s)						
Weight loss/appetite suppression	43/68 (63.2)	25/82 (30.5)	40/62 (64.5)	7/39 (17.9)	3/6 (50.0)	18/43 (41.9)
Improved glycemic control	40/68 (58.8)	68/82 (82.9)	35/62 (56.5)	29/39 (74.4)	5/6 (83.3)	39/43 (90.7)
Reduced insulin requirement	19/68 (27.9)	28/82 (34.1)	17/62 (27.4)	7/39 (17.9)	2/6 (33.3)	21/43 (48.8)
Improved glucose variability	8/68 (11.8)	22/82 (26.8)	8/62 (12.9)	11/39 (28.2)	0/6 (0.0)	11/43 (25.6)
No benefit	9/68 (13.2)	9/82 (11.0)	9/62 (14.5)	8/39 (20.5)	0/6 (0.0)	1/43 (2.3%)
Side effects perceived as related						
Yes	43/68 (63.2)	30/82 (36.6)	39/62 (62.9)	13/39 (33.3)	4/6 (66.7)	17/43 (39.5)
No	25/68 (36.8)	52/82 (63.4)	23/62 (37.1)	26/39 (66.7)	2/6 (33.3)	26/43 (60.5)
Side effect category						
GI symptoms	39/68 (57.4)	1/82 (1.2)	36/62 (58.0)	0/39 (0.0)	3/6 (50.0)	1/43 (2.3)
Hypoglycemia	2/68 (2.9)	0/82 (0.0)	2/62 (3.2)	0/39 (0.0)	0/6 (0.0)	0/43 (0.0)
Fatigue	2/68 (2.9)	1/82 (1.2)	1/62 (1.6)	0/39 (0.0)	1/6 (16.7)	1/43 (2.3)
Mycotic infection	0/68 (0.0)	9/82 (11.0)	0/62 (0.0)	5/39 (12.8)	0/62 (0.0)	4/43 (9.3)
UTI	0/68 (0.0)	8/82 (9.8)	0/62 (0.0)	3/39 (7.7)	0/62 (0.0)	5/43 (11.6)
DKA	0/68 (0.0)	4/82 (4.9)	0/62 (0.0)	2/39 (5.1)	0/6 (0.0)	2/43 (4.7)
Polyuria/dehydration	0/68 (0.0)	9/82 (11.0)	0/62 (0.0)	3/39 (7.7)	0/6 (0.0)	6/43 (14.0)
Pancreatitis	0/68 (0.0)	0/82 (0.0)	0/62 (0.0)	0/43 (0.0)	0/6 (0.0)	0/43 (0.0)
Duration of side effects						
<1 week	9/68 (13.2)	11/82 (13.4)	8/62 (13.0)	6/39 (15.4)	1/6 (16.7)	5/43 (11.6)
1–4 weeks	12/68 (17.6)	6/82 (7.3)	11/62 (17.8)	0/39 (0.0)	1/6 (16.7)	6/43 (14.0)
>4 weeks	16/68 (23.4)	8/82 (9.8)	14/62 (22.6)	4/39 (10.3)	2/6 (33.3)	4/43 (9.3)
Ongoing (at time of survey)	5/68 (7.3)	6/82 (7.3)	5/62 (8.1)	2/39 (5.1)	0/6 (0.0)	4/43 (9.3)
Current ongoing treatment^b^						
Yes	40/68 (58.8)	57/82 (69.5)	37/62 (59.7)	23/39 (59.0)	3/6 (50.0)	34/43 (79.1)
No	28/68 (41.2)	25/82 (30.5)	25/62 (40.3)	16/39 (41.0)	3/6 (50.0)	9/43 (20.9)
Reason for therapy discontinuation						
Side effects	14/68 (22.6)	9/82 (11.0)	12/62 (19.4)	6/39 (15.4)	2/6 (33.3)	3/43 (6.7)
Pregnancy/planning pregnancy	6/68 (8.8)	1/82 (1.2)	6/62 (9.7)	0/39 (0.0)	0/6 (0.0)	1/43 (2.3)
Insurance denial/cost	2/68 (2.9)	5/82 (6.1)	2/62 (3.2)	4/39 (10.3)	0/6 (0.0)	1/43 (2.3)
No benefit	4/68 (5.9)	5/82 (6.1)	4/62 (6.5)	4/39 (10.3)	0/6 (0.0)	1/43 (2.3)
Provider recommended	3/68 (4.4)	1/82 (1.2)	2/62 (3.2)	0/39 (0.0)	1/6 (16.7)	1/43 (2.3)
Willingness to reinitiate therapy (if not currently on therapy)						
Yes	17/28 (60.7)	14/25 (56.0)	16/25 (64)	8/16 (50)	1/3 (33.3)	6/9 (66.7)
Reason for wanting to reinitiate						
Glycemic control	14/17 (82.4)	10/14 (71.4)	13/16 (81.3)	4/8 (50)	1/1 (100)	6/6 (100)
Weight loss	6/17 (35.3)	2/14 (14.3)	6/16 (37.5)	1/8 (12.5)	0/1 (0.0)	1/6 (16.7)
Reduced insulin requirement	3/17 (17.6)	1/14 (7.1)	3/16 (18.8)	0/8 (0.0)	0/1 (0.0)	1/6 (16.7)
If provider recommended	3/17 (17.6)	0/14 (0.0)	3/16 (18.8)	4/8 (50)	0/1 (0.0)	0/6 (0.0)
No	11/28 (39.3)	11/25 (44.0)	9/25 (36)	8/16 (50)	2/3 (66.7)	3/9 (33.3)
Reasons for not wanting to reinitiate						
Side effects	8/11 (72.7)	5/11 (45.5)	6/9 (66.7)	3/8 (37.5)	2/2 (100)	2/3 (66.7)
No benefit	4/11 (36.4)	5/11 (45.5)	4/9 (44.4)	4/8 (50)	0/2 (0.0)	1/3 (33.3)
Insurance denial/cost	1/11 (9.1)	0/0 (0.0)	1/9 (11.1)	0/8 (0.0)	0/2 (0.0)	0/3 (0.0)

a Multiple answers were allowed (percentages for each question add to ⩾100%).

b As of the time of interview.

ASCVD, atherosclerotic cardiovascular disease; CKD, chronic kidney disease; DKA, diabetic ketoacidosis; GI, gastrointestinal; GLP-1RA, glucagon-like peptide 1 receptor agonist; *n*, number of participants who voiced the particular response; *N*, total number of participants who asked that question; SGLT2i, sodium glucose cotransporter 2 inhibitor; UTI, urinary tract infection.

### Perceived benefits attributed to adjuvant therapy

Most people (86.8% GLP-1RA and 89.0% SGLT2i users) reported ⩾1 benefit attributed to these therapies (Table 2). Among those treated with GLP-1RAs, the perceived benefits related to therapy were weight loss/appetite suppression (63.2%) and improved glycemic control (58.8%), while most of those treated with SGLT2i reported improved glycemic control (82.9%) and reduced insulin requirement (34.1%) as the main perceived benefits (Figure 1(b)).

Among those who indicated having initiated therapy with the goal of weight loss, more patients treated with GLP-1RA [91.4% (32/35)] reported achieving this goal, compared to those treated with SGLT2i [57.9% (11/19)]. In those who initiated therapy for improvement of glycemic control, a comparable percentage [83.3% (35/42) *versus* 88.1% (59/67)] reported achieving that benefit with both GLP-1RA and SGLT2i.

More participants in the Polish cohort reported any benefit from treatment with SGLT2i (97.7%) compared to those from the US cohort (79.5%) (Table 2). Specifically, more participants treated with SGLT2i in the Polish cohort compared to the US cohort reported improved glycemic control (90.7% *versus* 74.4%), weight loss/suppressed appetite (41.9% *versus* 17.9%), or reduced insulin requirement (48.8% *versus* 17.9%), and only 2.3% of participants in the Polish cohort reported no benefits *versus* 20.5% in the US cohort.

### Side effects perceived to be related to adjuvant therapy

Side effects perceived to be related to the therapy were more commonly reported by GLP-1RA *versus* SGLT2i users (63.2% *versus* 36.6%, respectively). Gastrointestinal symptoms were the most reported side effects among GLP-1RA users (57.4% *versus* 1.2% of SGLT2i users; Figure 1(c)). Overall, among SGLT2i users, 11.8% reported genital mycotic infections, 9.0% reported urinary tract infections (UTIs), and 11% reported polyuria/dehydration. DKA was reported by 4.9% SGLT2i users and no GLP-1RA user. No episodes of pancreatitis were reported. Of the 43 GLP-1RA users who reported side effects, less than a third (32.3%) discontinued therapy because of the side effects (22.6% of all GLP-1RA users). For SGLT2i users, 9/30 patients (30%) who experienced side effects discontinued because of the reported side effects (11% of all SGLT2i users). Among those treated with SGLT2i in Poland *versus* United States, the nature and frequency of the reported side effects were comparable.

### Self-reported reasons for adjuvant therapy discontinuation

At the time of the survey 58.8% of GLP-1RA users and 69.5% of SGLT2i users were still on therapy. For all GLP-1RA users, the most common reasons for discontinuing therapy were side effects (22.6%), pregnancy/planning pregnancy (8.8%), and no benefit from therapy (5.9%). SGLT2i users discontinued treatment due to side effects (11.0%), insurance denial/cost (6.1%), and no observed benefit from therapy (6.1%) (Figure 1(d)).

Among those treated with SGLT2i, more participants reported having discontinued therapy in the United States *versus* Poland (41% *versus* 20.9%), with the top reasons for discontinuing SGLT2i in the United States being side effects (15.4%), insurance denial (10.3%), and no benefit (10.3%) (Table 2).

### Patient-reported willingness to reinitiate adjuvant therapy (if not actively using at the time of the interview)

Of those not currently on therapy, 60.7% of prior GLP-1RA users and 56.0% of prior SGLT2i users were willing to reinitiate the respective therapy. The reasons for reinitiation were reported to be glycemic control (82.4% *versus* 71.4% for the GLP-1RA *versus* SGLT2i users) and weight loss (35.3% *versus* 14.3%). For those unwilling to restart therapy, the main reasons for this were side effects (72.7% *versus* 45.5% for the GLP-1RA *versus* SGLT2i users) and no perceived benefit (36.4% *versus* 45.5%).

## Discussion

We evaluated patient-reported subjective experiences with adjuvant therapies (GLP1-RA and SGLT2i) for T1DM. We found that most patients using either class reported at least one benefit. For both groups, the most common reason to initiate therapy was improved glycemic control, though more patients reported starting GLP-1RA therapy for weight loss compared to SGLT2i. More GLP-1RA users reported weight loss as a treatment-related benefit, while more SGLT2i users reported improved glycemic control as a treatment-related benefit. Side effects were more common among GLP-1RA users, and slightly more GLP-1RA users discontinued treatment due to side effects compared to SGLT2i users. There were no reports of pancreatitis in this cohort, but DKA occurred in 4.9% of SGLT2i users (none among GLP-1RA users). Most patients were still on therapy at the time of survey. For those who stopped treatment, most were willing to reinitiate therapy, primarily for the perceived benefits of glycemic control and weight loss.

This study provides a novel and unique perspective regarding the reasons for initiation, perceived benefits, and side effects perceived to be related to therapy obtained directly from a real-world cohort of users of adjuvant therapy for T1DM at a US and a European academic institution. Previous reports from real-world cohorts reported on objective outcomes, like HbA1c, weight, or insulin dose. A retrospective study by Palanca *et al*.^18^ evaluated 199 adult patients with T1DM treated with SGLT2i adjuvant therapy at two European centers. After 12 months of therapy there was a 0.5% reduction in HbA1c, 2.9 kg weight loss, and 8.5% reduction in daily insulin use. Another study by Seufert *et al*.^19^ also reported on the use of SGLT2i in 233 patients with T1DM and showed significant decreases in HbA1c (−0.63%), total cholesterol (−8.70 mg/dl), and low density lipoprotein (LDL) cholesterol (−5.58 mg/dl) after 12 months on therapy. There are very few reports on the real-world outcomes with GLP-1RA. Harrison *et al*.^20^ reported on 11 patients treated with exenatide or liraglutide and found that after 10 weeks of therapy, there was a significant decrease in weight (4.2% of total body weight), total daily dose of insulin (19.2% reduction), and HbA1c (0.4% reduction) which was maintained at 20 weeks of follow-up. Other retrospective studies with GLP-1RA use in T1DM showed similar findings of reductions in HbA1c, weight, and insulin dose, although the number of patients in those studies were similarly small (11 patients in 1 study and 27 in the other).^21,22^ Most recently, Edwards *et al*.^16^ published data on real-world use of SGLT2i and GLP-1RA, this study reported on the largest real-world cohort of GLP-1RA use in T1DM. They reported statistically significant reductions, compared to baseline, in weight (−5.1 kg), HbA1c (−0.4%) and total daily insulin required (−19.9 units) after 1 year of treatment with GLP-1RAs, while SGLT2i users experienced significant reductions in HbA1c (−0.7%) and basal insulin (−5.7 units). While these reports provided important information about real-world outcomes of treatment with GLP-1RA and SGLT2i in this population, none of these studies assessed the users’ perspectives and experiences with these treatments. Patient-reported outcomes were collected in the randomized clinical trials which evaluated liraglutide use in T1DM. In both studies (ADJUNCT-1 and ADJUNCT-2), those treated with liraglutide reported significantly improved quality of life compared to placebo as per the Treatment-Related Impact Measure for Diabetes (TRIM-D) survey.^7,8^ Similarly, in the inTandem1 and inTandem2 studies, those randomized to sotagliflozin reported significant improvement in treatment satisfaction, and significant reductions in diabetes distress compared to placebo as per the Diabetes Treatment Satisfaction Questionnaire and the Diabetes Distress Screening Scale 2.^23,24^ These data provide an important assessment of quality of life improvements with these treatments, but such findings might not translate to real-world practice. Understanding patients’ perceptions regarding these therapies can strengthen the patient–provider relationship, guide how providers may use these medications for improving outcomes, and ensure patient compliance with these therapies.

In our cohort, most patients initiated these therapies to improve glycemic control. This suggests that despite advancements in insulin preparations and diabetes technology, patients with T1DM still have an interest in using additional adjuvant therapies to improve glycemic control. Importantly, most patients reported benefits perceived to be related to therapy and were willing to continue therapy, or reinitiate if they were not currently on therapy. This indicates a high level of satisfaction with such treatments, an especially notable finding given the relatively long duration of treatment of these participants.

While there have not been prior studies involving interviews and patient-reported outcomes with GLP-1RA use, Ervin *et al*.^25^ conducted exit interviews with 42 participants with T1DM who took part in the phase III sotagliflozin trials. Similar to our findings, participants reported joining these trials with hopes to improve lack of stable blood sugar control, lower their HbA1c, and reduce weight. More patients on treatment *versus* placebo reported fewer hyper- and hypoglycemic events, improvements in weight, and reduced insulin requirements.

Regarding safety concerns in our cohort, there were no reported cases of pancreatitis among GLP-1RA users, in-line with existing data that confirms the lack of excess risk of pancreatitis in the setting of treatment with GLP-1RA,^26^ and similar to our recently reported clinical outcomes data.^16^ On the other hand, DKA was reported by 4.9% of SGLT2i users, higher than the reported incidence of 3.5% in a recent real-world report of patients with T1DM using SGLT2i,^18^ but lower than our clinical outcomes data which showed that 12.8% of SGLT2i users had a DKA event over a median follow-up duration of 29.5 months.^16^ Of note, only 39.4% of the cohort interviewed for this report also contributed clinical data to our previously published work.^15^ Overall, DKA events reported by participants in this report were higher than those reported in the controlled clinical trials (2.2–4.3%) where more careful patient selection occurs along with very intensive monitoring.^27^ This stresses the importance of careful patient selection when considering such treatments in real-world practice, detailed education on how to manage specific health situations including days with low or no oral intake, how to manage insulin treatment, and how to recognize early the signs/symptoms of DKA, particularly in the setting of euglycemic DKA where blood glucose may not be elevated. UTIs and mycotic infections were reported by 9.0% and 11.8% of SGLT2i users, respectively – which was in the range of those reported in other T1DM clinical trials with SGLT2i (UTI: 1.6–11.6%; mycotic infections: 6.4–15.5%),^10−12^ though higher than that reported in clinical trials involving patients with T2DM using SGLT2i (usually < 10%).^28^ Patients should be regularly counseled on good hygiene and adequate hydration while on SGLT2i therapy to minimize such complications.

At the Polish site, SGLT2i use was vastly more prevalent *versus* GLP-1RA use, likely because SGLT2i were approved for T1DM management in Europe (both sotagliflozin and dapagliflozin were on label at the time the surveys were conducted), while GLP-1RA are off label and not covered by insurance. While there were very few GLP-1RA users at the Polish site to contrast their experience to that of their US counterparts, there were several notable differences when comparing SGLT2i users across the two sites. Interestingly, the reported reasons for initiating therapy were different. Polish SGLT2i users initiated therapy more frequently for weight loss/appetite suppression than their US counterparts, likely in keeping with the on-label indication for use of SGLT2i in patients with BMI ⩾ 27.0 and suboptimal control on insulin and the fact that in the United States those interested in weight loss were likely preferentially offered a GLP-1RA rather than a SGLT2i. Also, the Polish patients were more likely to report a decrease in insulin dose as a reason for initiation of SGLT2i, while US patients were more likely to report either a decrease in glucose variability or cardiorenal benefits as reason for initiation of SLGT2i. Polish SGLT2i users more frequently perceived benefits related to therapy, specifically weight loss/appetite suppression, improved glycemic control, and reduced insulin requirement compared to their US counterparts. This may be due to selection of a patient population more likely to benefit from SGLT2i therapy given its label indications in Europe. Previous real-world data has shown that patients with BMI ⩾ 27.0 had greater weight loss compared to those with BMI ⩽ 27.0 and greater HbA1c reductions in those with HbA1c ⩾ 8.0% compared to HbA1c ⩽ 8.0%.^18^ Rates of DKA and UTI/mycotic infections were the same across both US and Polish SGLT2i users, though Polish SGLT2i users reported more polyuria/dehydration. Given that Polish SGLT2i users reported benefits more frequently with therapy, it is not surprising that a greater proportion of those users were still on therapy at the time of interview. US SGLT2i users more frequently reported discontinuing therapy due to side effects or no benefit from therapy than their Polish counterparts. These findings highlight that this therapy should be used in carefully selected patients for whom the benefits will outweigh the risks. Given that SGLT2i therapy is off label in the United States for patients with T1DM, it was not unexpected that more US participants reported discontinuing therapy due to cost or insurance denials. Despite SGLT2i being approved for T1DM in Europe at the time of survey, the Polish cohort had a much shorter median duration of therapy. This may relate to the fact that in the United States, SGLT2i use was entirely off label and may have started around the time of first SGLT2i approval in 2013. In Poland however, the majority of SGLT2i use may have only started around 2019 when these agents were approved for T1DM, thus leading to a shorter duration of therapy in the Polish cohort.

Several study limitations are noteworthy. The purpose of our study was to determine patient-reported experiences with these medications, and there was no objective measure of these reported positive outcomes or adverse events, such as DKA, with the data collected then subject to recall bias. While the study population represents two large academic centers on two continents, the population is not representative of all those with T1DM who use adjuvant therapies. We had a positive response rate of only approximately 52%, which potentially can be a source of bias in our survey sample. Furthermore, our population was overwhelmingly White and non-Hispanic, thus caution is advised regarding generalizability to other groups. Selection bias was introduced by restricting the eligible population to those who received treatment for a minimum of 90 days. While this criterion increased the likelihood of recalling accurate information about the therapy and allowed us to characterize the perceived benefits and drawbacks of chronic therapy, the discontinuation rates are likely underestimated by excluding those who stopped therapy early. The Polish cohort had very few GLP-1RA users, thus limiting our ability to compare experiences across institutions among GLP1-RA users. We used an unvalidated open-ended survey because a topic-specific standardized and validated survey tool does not exist. Furthermore, we used a small number of specific and directed questions to minimize the time commitment for the participants and thus increase the likelihood of response.

## Conclusion

Patients with T1DM who use adjuvant therapies with GLP-1RA or SGLT2i report observing benefits related to treatment. While significant numbers of patients may experience side effects (particularly with GLP-1RA use), most patients are able to continue therapy and are willing to reinitiate if not currently treated. Understanding patients’ perspectives and experiences with these agents is an important step in providing personalized care, understanding patient-centric treatment goals and priorities, and increasing compliance with a treatment plan. However, careful patient selection and monitoring is critical to minimize side effects and maximize benefits, especially as these therapies are largely used off label.

## Declarations

**Ethics approval and consent to participate:** This project was approved by the Institutional Review Boards of the UT Southwestern and Poznan University of Medical Sciences (reference STU 2021-0145). Informed consent was waived for the retrospective chart review and identification of patients who met criteria for survey participation. At the time of telephone survey, participants provided verbal consent to the telephone interview prior to survey questions being asked. Participants were made aware that if any question made them uncomfortable, they could rescind consent at any time during the survey and results would not be used or published. Participants were aware that the reported survey information would be anonymous. Verbal consent was recorded in REDCap by the surveyor.

**Consent for publication:** Not applicable.

**Authors’ note:** Prior Publications: Data from this study were submitted and presented as an abstract and poster at the American Association of Clinical Endocrinology (AACE) Annual Meeting 2022 held on 12–14th May 2022 in San Diego, California, USA.

**Availability of data and materials:** Not applicable
